# Synthesis of Pyridazine Derivatives by Suzuki-Miyaura Cross-Coupling Reaction and Evaluation of Their Optical and Electronic Properties through Experimental and Theoretical Studies

**DOI:** 10.3390/molecules23113014

**Published:** 2018-11-18

**Authors:** Sara S. M. Fernandes, João Aires-de-Sousa, Michael Belsley, M. Manuela M. Raposo

**Affiliations:** 1 Centro de Química, Universidade do Minho, Campus de Gualtar, 4710-057 Braga, Portugal; sarasmfernandes@gmail.com; 2 LAQV and Departamento de Química, Faculdade de Ciências e Tecnologia, Universidade Nova de Lisboa, 2829-516 Caparica, Portugal; jas@fct.unl.pt; 3 Centro de Física, Universidade do Minho, Campus de Gualtar, 4710-057 Braga, Portugal; belsley@fisica.uminho.pt

**Keywords:** Suzuki-Miyaura coupling, pyridazine, Second Harmonic Generators (SHG), Nonlinear optics (NLO), Density Functional Theory (DFT)

## Abstract

A series of π-conjugated molecules, based on pyridazine and thiophene heterocycles **3a**–**e**, were synthesized using commercially, or readily available, coupling components, through a palladium catalyzed Suzuki-Miyaura cross-coupling reaction. The electron-deficient pyridazine heterocycle was functionalized by a thiophene electron-rich heterocycle at position six, and different (hetero)aromatic moieties (phenyl, thienyl, furanyl) were functionalized with electron acceptor groups at position three. Density Functional Theory (DFT) calculations were carried out to obtain information on the conformation, electronic structure, electron distribution, dipolar moment, and molecular nonlinear response of the synthesized push-pull pyridazine derivatives. Hyper-Rayleigh scattering in 1,4-dioxane solutions, using a fundamental wavelength of 1064 nm, was used to evaluate their second-order nonlinear optical properties. The thienylpyridazine functionalized with the cyano-phenyl moiety exhibited the largest first hyperpolarizability (*β* = 175 × 10^−30^ esu, using the T convention) indicating its potential as a second harmonic generation (SHG) chromophore.

## 1. Introduction

Few reactions have contributed to enhancing the efficiency of organic synthesis as much as the palladium-catalyzed cross-couplings. The reactions are used in research worldwide as well as in the commercial production of pharmaceuticals and a variety of molecules are utilized in the electronics industry, amongst other examples.

Among a diverse number of palladium-catalyzed cross couplings, the Suzuki-Miyaura cross-coupling reaction offers, at present, one of the most efficient ways to prepare π-conjugated heterocyclic systems through the formation of carbon–carbon bonds. Suzuki coupling is a versatile method of synthesis, possessing a large number of advantages. It employs readily available reagents (about 1800 compounds: Boronic acids, boronate esters, etc.) that are commercially available, it occurs under mild reaction conditions, it is largely unaffected by the presence of water, it tolerates a wide variety of functional groups, it generally proceeds regio- and stereoselectively, while the inorganic by-products are non-toxic and easily removed from the reaction mixture. All of these qualities contribute to make the Suzuki coupling suitable not only for laboratories, but also for myriad industrial processes [1,2,3,4,5,6,7].

Recently, research targeting molecules based on pyridazine heterocycles has grown considerably due to their numerous applications as therapeutic agents [8,9], chemiluminescent materials [10,11,12], ligands for heterogeneous catalysis [13,14], in supramolecular chemistry [15], and nonlinear optical [16,17,18,19,20,21,22,23,24] and photovoltaic materials [25]. This wide array of interesting applications has motivated the development of procedures for the preparation of differently substituted pyridazines. In general, two methods are reported which consist of: (i) Cyclisation reactions using 1,4-dicarbonilic precursors and hydrazine [26,27,28,29,30,31,32], in order to construct the pyridazine ring or (ii) by functionalization through nucleophilic substitution [33,34,35,36,37,38], ortho-metallation [39,40,41] and cross-couplings reactions (Suzuki, Stille, Sonogashira, etc.) [42,43,44,45,46,47,48,49,50] or a combination of cyclisation and cross-coupling reactions [51].

The synthesis of pyridazine derivatives through palladium cross-coupling reactions is quite advantageous compared to cyclisation methodology. This is due to the ready availability of the coupling components as well as the versatility of this synthetic method. Additionally, the electronic deficient nature of the pyridazine heterocycle facilitates the oxidative addition of palladium to a halogen–carbon bond without the use of specific and expensive ligands [18,46]. This is a great advantage given the widespread commercial availability of halogenated pyridazine derivatives, or alternatively, their relatively easy preparation.

Whereas several series of pyridazine derivatives have been reported as efficient two-photon absorption (TPA) fluorophores [18,20,23], studies describing nonlinear optics (NLO) second harmonic generation properties (SHG) remain limited to a restricted number of compounds [16,19,21,22,24], which included organometallic compounds bearing pyridazine ligands [21]. Furthermore, it is noteworthy fact that none of these publications report the synthesis and evaluation of SHG pyridazine NLOphores constituted by pyridazine and thiophene heterocyclic moieties, which act simultaneously as π-bridge and auxiliary electron acceptor or electron donor groups, respectively.

Among the many classes of π-conjugated organic systems, donor-acceptor substituted heterocyclic compounds are of great interest because it has been experimentally and theoretically demonstrated that they increase the first hyperpolarizabilities of push-pull chromophores with respect to aryl analogues. The incorporation of different heterocycles into the π-conjugated systems can be a powerful approach for tuning the system to obtain specific optoelectronic properties. The heterocycles convey higher polarizability, modulate the conjugation pathway, promote thermal and chemical robustness, and behave as auxiliary electron donors/acceptors as well as π-bridges, in addition to serving as components for further modification. It is well known that electron-rich heterocycles such as thiophene and pyrrole, and electron-deficient heterocycles such as azole and azine and diazine derivatives: (Benzo)thiazole benzothiadiazole, pyridine, pyridazine etc. can act simultaneously as π-bridges and electron donors or acceptors, respectively [16,17,18,19,20,21,22,23,24,38,52,53,54,55,56,57,58,59,60,61,62,63].

During the past 14 years, our research group has reported several series of push-pull π-conjugated heterocyclic compounds bearing electron-deficient azines (pyridine, quinoline, phenanthroline), diazines (pyridazine, phthalazine) or azoles (thiazole, benzothiazole, benzimidazole and benzothiadiazole). These systems have applications as SHG chromophores [52,53,54,55,56,57,58,59,60,61,62,63], organic sensitizers for dye sensitized solar cells (DSSCs), organic fluorophores for organic light emitting diodes (OLEDs), optical chemosensors, DNA intercalations, heterogeneous catalysts, etc.

In the present work we intended to employ the electron-rich thiophene heterocycle as an auxiliary electron donor group, and simultaneously as an efficient π-bridge. Alternatively, pyridazine, being an electron deficient heterocycle, linked directly to the aryl or heteroaryl moieties functionalized with the acceptor group can play the dual roles of auxiliary acceptor group and π-bridge connector.

Based on the above arguments, we were motivated to expand our investigation, concerning push–pull heterocyclic systems as SHG NLOphores, to a series of six new thienylpiridazine derivatives which were synthesized through palladium catalyzed Suzuki-Miyaura coupling, using a 3-bromo-6-(thiophen-2-yl)pyridazine [51] derivative and several hetero(aromatic) boronic acids as coupling components. The new pyridazine NLOphores were functionalized with an electron-rich thiophene heterocycle as electron donor group/π-spacer, and a hetero(aryl) moiety functionalized with several groups with different electron ability. This research was conducted with the purpose of studying the relationship between their structure and second harmonic generation efficiency.

## 2. Results and Discussion

### 2.1. Synthesis and Characterization

A new series of six thienylpyridazine derivatives **3a**–**e** were synthesized through palladium catalyzed Suzuki–Miyaura coupling, using 3-bromo-6-(thiophen-2-yl)pyridazine **2** [51] as the halogenated coupling component, which was prepared by the reaction of thienylpyridazinone **1** with POBr_3_. On the other hand, compound **2** was prepared by Friedel-Crafts acylation of thiophene with 3-carbomethoxypropionyl chloride, giving methyl 4-(2-thienyl)-4-oxobutanoate. This precursor was subsequently cyclized giving the thienylpyridazinone **2** [51,64,65] by condensation with hydrazine hydrate in ethanol at reflux.

**Scheme 1:**
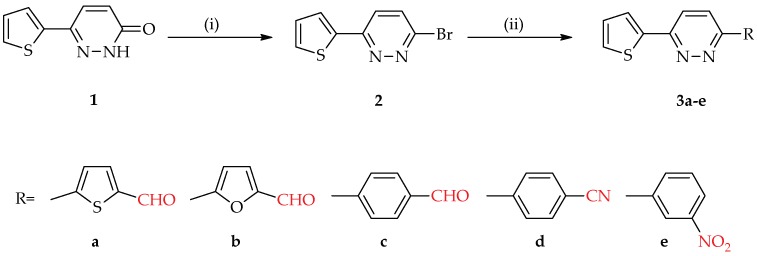
Synthesis of thienylpyridazines **2**–**3**: (i) POBr_3_, Δ; (ii) DME (1,2-dimethoxyethane), R-B(OH)_2_, Ethanol, Pd(PPh_3_)_4_, Na_2_CO_3_ 2 M, 80 °C, N_2_.

The final push-pull thienylpyridazine derivatives **3a**–**e** were synthesized by Suzuki-Miyaura cross-coupling reaction of 3-bromo-6-(thiophen-2-yl)pyridazine **2** with commercially available (hetero)aryl-boronic acids in fair-to-low yields (14% to 28%) (Table 1, Scheme 1).

The low yields of compounds **3a**–**e** might be explained by the possible homocoupling of the brominated thienylpyridazine precursor **2**, or the competitive hydrolytic deboronation of the hetero(aryl) boronic acids, especially because the hetero(aryl)boronic acids bears electron-attracting groups [43,45]. Due to instability, the hydrolysis of the C-Br bond of the thienylpiridazine **2** could also occur, giving the precursor pyridazinone **2** [51]. The novel compounds were characterized by standard spectroscopic techniques.

### 2.2. Study of the Optical (Linear and Nonlinear) Properties

**Figure 1:**
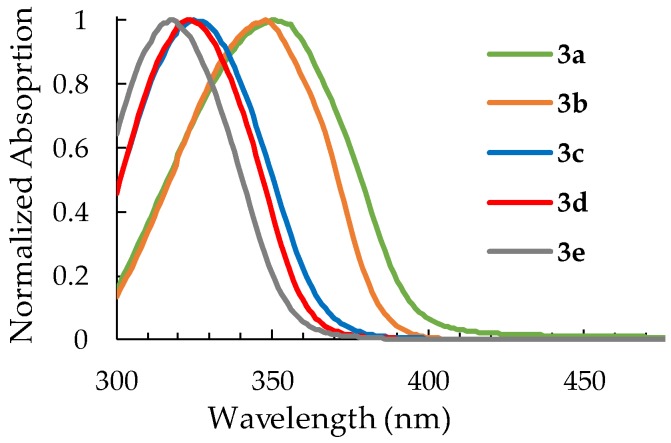
Normalized absorption spectra of compounds **3a**–**e** in ethanol: **3a** λ_max_ = 357 nm; **3b** λ_max_ = 354 nm; **3c** λ_max_ = 332 nm; **3d** λ_max_ = 323 nm; **3e** λ_max_ = 314 nm.

The linear optical properties of thienylpyridazines **3a**–**e** were studied in ethanol at room temperature (Table 1, Figure 1). All compounds exhibited a strong and broad absorption band with high molar extinction coefficients in the range of 24,100 to 29,800 M^−1^·cm^−1^ with maximum absorption peaks found between 314 and 357 nm. The variation of the maximum absorption wavelength amongst the thienylpyridazine derivatives depended on the electronic nature of the spacer and the electron withdrawing moieties, thus bathochromic shifts were found when substituting the phenyl ring for the furan (22 nm) or thiophene (25 nm) heterocycles due to the increase in the electron donating ability, and when substituting the nitro group in the meta position for the cyano (9 nm) or formyl (18 nm) groups in the para position. This was due to the electron withdrawing strength of these groups.

The fluorescence properties of thienylpyridazines **3a**–**e** were studied by exciting the compounds at the wavelength of maximum absorption, at room temperature (Table 1). All thienylpyridazines showed very weak emissive properties; with relatively low quantum yields in the range of 0.003 to 0.006. Given that the molar extinction coefficients are all greater than 24,000 M^−1^·cm^−1^, indicating that the transitions have reasonable oscillator strength, the low quantum yields are likely to be an indication of strong quenching, perhaps induced by hydrogen bonding of the solvent with the nitrogen atoms [68]. However, the molar extinction coefficients of compounds **3d** and **3e** were identical whether dissolved in ethanol (Table 1) or 1,2-dioxane (Table 2). Furthermore, any strong excited state quenching is unlikely to affect greatly the second order nonlinear response of these molecules, which is produced dominantly by virtual transitions, at least in the absence of any multiphoton absorption.

The hyper-Rayleigh scattering (HRS) technique was used to determine the first hyperpolarizabilities *β* of thienylpyridazines **3a**–**e**, at a fundamental wavelength of 1064 nm [69,70]. The hyperpolarizabilty *β* values were measured against a reference solution of *p*-nitroaniline (*p*NA), using 1,4-dioxane as a solvent [71,72]. Proper care was taken to account for possible fluorescence of the thienylpyridazines **3a**–**e** [73].

The static hyperpolarizabilty *β*_0_ values [74,75,76] for the thienylpyridazines **3a**–**e** showed the same trend as the measured values, however these values are only indicative and should be treated with discretion, as they were calculated using a simple two-level model neglecting damping.

The data in Table 2 show an enhancement of the SHG response with an increase of the auxiliary electron donating ability of the spacer upon changing the phenyl ring (*β* = 54 × 10^−30^ esu for **3c**) with furan heterocycle (*β* = 100 × 10^−30^ esu for **3b**), and then for a thiophene moiety (*β* = 155 × 10^−30^ esu for **3a**). For compound **3e**, with the nitro group at the *meta* position, it was not possible to quantify the SHG signal due to interference resulting from competing fluorescence from multiphoton absorption. The highest measured hyperpolarizability value was achieved by thienylpyridazine **3d** having a phenyl ring substituted at position four with a cyano group (*β* = 175 × 10^−30^ esu).

### 2.3. Theoretical Calculations

**Figure 2:**
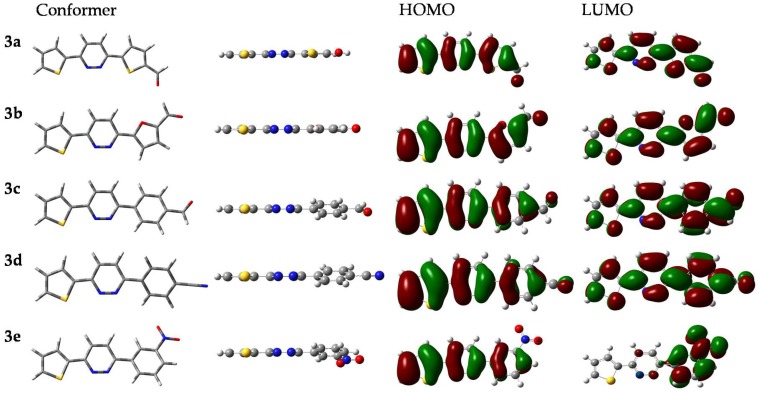
Optimized geometry of the lowest energy conformer, and frontier molecular orbitals of thienylpyridazines **3a**–**e** (HOMO: Highest Occupied Molecular Orbital; LUMO: Lowest Unoccupied Molecular Orbital).

Density functional theory (DFT) calculations were performed to understand the structural differences and the variation of the electronic properties of these thienylpyridazines, and to establish a comparative computational basis for this series. The dipole moments and hyperpolarizabilities *β* were calculated for thienylpyridazines **3a**–**e**, as well as the energy levels and the respective electron density maps that were computed in a polarized solvent continuum of 1,4-dioxane. The results are shown in Table 3 and Figure 2.

Each thienylpyridazine derivative can exist as several different conformers, depending on the relative arrangement of their components. We present the lowest energy forms, which are responsible for the calculated properties. Coplanarity was observed between the three rings in molecules **3a** and **3b**, while the replacement of a substituted thiophene by a functionalized phenyl ring reduced the planarization of the conjugated system in molecules **3c**–**e**.

The maps of frontier orbitals showed diffuse and overlapping highest occupied molecular orbital (HOMO) and lowest unoccupied molecular orbital (LUMO) densities, the HOMO density slightly more concentrated on the thiophene donor group and the LUMO density slightly more concentrated on the (hetero)aromatic acceptor moiety, except in chromophore **3e**. In **3e** the LUMO was essentially localized on the meta-nitrophenyl acceptor group. No significant correlation was observed between HOMO-LUMO gaps and maxima of absorption spectra.

The estimated dipole moments for the five molecules range between 4.3 and 8.9 Debye (in 1,4-dioxane), and exhibit a higher correlation with the experimentally determined hyperpolarizabilities than the calculated hyperpolarizabilities. However, the moderate experimental *β* values are in good agreement with their calculated small values.

## 3. Experimental

### 3.1. Materials and Methods

Phosphorous (V) oxybromide and boronic acids were procured from Sigma Aldrich Chemie, Steinheim, Germany and Acros Organics, Geel, Belgium. Other commercial reagents (NaCl, NaOH, quinine sulfate, ammonia, MgSO_4_, Na_2_CO_3_, Pd(PPh_3_)_4_), and solvents (dimethoxyethane, ethanol, dichloromethane, chloroform, *n*-hexane, dioxane, light petroleum (40–60 °C), acetone-*d*_6_) were obtained from Panreac Quimica S.L.U., Barcelona, Spain) and were used without further purification. The progress of the reaction was checked by means of thin layer chromatography on 0.25 mm thick precoated silica plates (Merck Fertigplatten Kieselgel 60 F254; Merck, Darmstadt, Germany); and the spots were visualized using UV light. Silica gel column chromatography (Merck Kieselgel, 230 to 400 mesh; Merck, Darmstadt, Germany) was used in the purification of the compounds. NMR spectra were performed on a BruckerG Avance II 400 (Bruker Daltonics, Bremen, Germany), working frequency of 400 MHz for ^1^H and 100.6 MHz for ^13^C, and the solvent peak was used as internal reference. The solvents are specified in parenthesis before the chemical shifts values (*δ* relative to tetramethylsilane (TMS)—tetramethylsilane). Peak assignments were obtained by comparison of chemical shifts, peak multiplicities, and *J* values, and were sustained by spin decoupling-double resonance and bidimensional heteronuclear HMBC (Heteronuclear Multiple Bond Correlation) and HMQC (Heteronuclear Multiple-Quantum Correlation) techniques. Infrared spectra were obtained on a BOMEM MB 104 spectrophotometer (BOMEM, Québec, QC, Canada). UV-vis absorption spectra were recorded with a Shimadzu UV/2501PC spectrophotometer (Shimadzu Coorporation, China). Fluorescence spectra were obtained with a FluoroMax-4 spectrofluorometer (Horiba Jobin Yvon, Edison, New Jersey, USA). Luminescence quantum yields were obtained in comparison with a solution of quinine sulfate in 0.05 M H_2_SO_4_ as standard and corrected for the refraction index of the solvents [66,67]. Melting points were determined on a Gallenkamp machine (Gallenkamp, UK). Mass spectrometry analyses were performed at the C.A.C.T.I.—Unidad de Espectrometria de Masas of the University of Vigo, Spain (Bruker Daltonics, Bremen, Germany).

### 3.2. Synthesis and Characterization

#### 3.2.1. Procedure for the Synthesis of Thienylpyridazine Precursor **2**

A mixture of 6-(thiophen-2-yl)pyridazin-3(2*H*)-one **1** (2.8 mmol, 0.5 g) and POBr_3_ (5.5 mmol, 1.6 g) was heated for 6 h at 110 to 120 °C. The mixture was cooled till room temperature and then poured onto ice-water, basified with a solution of ammonia (2 M), and stirred for 30 min to give a brown solid. The suspension was filtered and the solid washed with water and light petroleum to give the pure thienylpyridazine **2** as brown solid (76%). ^1^H-NMR (Acetone-*d*_6_, 400 MHz) *δ* 7.26 (dd, 1H, H-4′, *J* = 5.2 Hz, *J* = 3.6 Hz), 7.76 (dd, 1H, H-5′, *J* = 5.2 Hz, *J* = 1.2 Hz), 7.94 (dd, 1H, H-3′, *J* = 3.6 Hz, *J* = 1.2 Hz), 7.96 (d, 1H, H-5, *J* = 9.2 Hz), 8.14 (d, 1H, H-4, *J* = 9.2 Hz) ppm.

#### 3.2.2. General procedure for the Synthesis of Thienylpyridazines **3a**–**e** through Suzuki-Miyaura Cross Coupling

3-Bromo-6-(thiophen-2-yl)pyridazine **2** (0.5 mmol) was coupled with the appropriate (hetero)aromatic boronic acids (0.6 mmol) in a mixture of DME (8 mL), ethanol (2 mL), aqueous 2 M Na_2_CO_3_ (1 mL), and Pd(PPh_3_)_4_ (5 mol %) at 80 °C, under nitrogen. The reaction time (48 h) was determined by thin layer chromatography (TLC). The reaction mixture was extracted, after cooling, with chloroform (3 × 20 mL) followed by extraction with a saturated solution of NaCl (20 mL). After the separation of the phases, the organic layer was washed with water (3 × 10 mL) and with a solution of NaOH (10%) (10 mL). The organic phase obtained was dried (MgSO_4_), filtered, and the solvent removed, giving a crude mixture which was purified using silica gel column chromatography and mixtures of dichloromethane in light petroleum of (40–60 °C) increasing polarity. Evaporation of the solvent gave the coupled products as solids that were recrystallized from dichloromethane/hexane giving the pure pyridazines **3a**–**e**.

5-(6′-Thiophen-2″-yl)pyridazin-3′-yl)thiophene-2-carbaldehyde **3a**. Light brown solid (28%). Mp: 247–250 °C. UV (ethanol): λ_max_ nm (*ε*, M^−1^·cm^−1^) 357, (29,790). IR *ν* 1,657 (C=O) cm^−1^. ^1^H-NMR (Acetone-*d*_6_, 400 MHz) *δ* 7.28 (dd, 1H, H-4″, *J* = 5.2 Hz, *J* = 3.6 Hz), 7.77 (dd, 1H, H-5″, *J* = 5.2 Hz, *J* = 1.2 Hz), 7.98 (dd, 1H, H-3″, *J* = 3.6 Hz, *J* = 1.2 Hz), 8.06–8.10 (m, 2H, H-3, H-4, *J* = 0.8 Hz), 8.27 (d, 1H, H-5′, *J* = 9.2 Hz), 8.33 (d, 1H, H-4′, *J* = 9.2 Hz), 10.06 (s, 1H, CHO) ppm. ^13^C-NMR (Acetone-*d*_6_, 400 MHz) *δ* 123.6, 124.5, 128.1, 128.2, 129.2, 130.9, 138.3, 141.4, 146.2, 150.0, 153.6, 155.5, 184.5 ppm. MS (EI) *m*/*z* (%) = 273 ([M + 1]^+^, 6), 272 ([M]^+^, 35), 244 (3), 135 (12), 108 (100). HRMS: *m*/*z* (EI) for C_13_H_8_N_2_OS_2_; calcd 272.0078; found 272.0073.

5-(6′-(Thiophen-2″-yl)pyridazin-3′-yl)furan-2-carbaldehyde **3b.** Yellow solid (14%). Mp: 207–210 °C. UV (chloroform): λ_max_ nm (*ε*, M^−1^·cm^−1^) 354 (27,790). IR *ν* 1,664 (C=O) cm^−1^. ^1^H-NMR (Acetone-*d*_6_, 400 MHz) *δ* 7.29 (dd, 1H, H-4″, *J* = 4.9 Hz, *J* = 3.6 Hz), 7.58 (d, 1H, H-4, *J* = 3.9 Hz), 7.68 (d, 1H, H-3, *J* = 3.9 Hz), 7.78 (dd, 1H, H-5″, *J* = 4.9 Hz, *J* = 0.9 Hz), 8.00 (dd, 1H, H-3″, *J* = 3.6 Hz, *J* = 1.2 Hz), 8.18 (d, 1H, H-4′, *J* = 8.7 Hz), 8.31 (d, 1H, H-5′, *J* = 9.3 Hz), 9.81 (s, 1H, CHO) ppm. ^13^C-NMR (Acetone-*d*_6_, 400 MHz) *δ* 112.4, 123.5, 124.2, 128.3, 129.3, 131.0, 141.4, 150.6, 154.5, 155.2, 156.3, 178.6 ppm. MS (EI) *m*/*z* (%) = 257 ([M + 1]^+^, 6), 256 ([M]^+^, 78), 228 (6), 171 (12), 108 (100). HRMS: *m*/*z* (EI) for C_13_H_8_N_2_O_2_S; calcd 256.0306; found 256.0302.

4-(6′-(Thiophen-2″-yl)pyridazin-3′-yl)benzaldehyde **3c**. Yellow solid (15%). Mp: 214–216 °C. UV (ethanol): λ_max_ nm (*ε*, M^−1^·cm^−1^) 332 (25,990). IR *ν* 1,639 (C=O) cm^−1^. ^1^H-NMR (Acetone-*d*_6_, 400 MHz) *δ* 7.29 (dd, 1H, H-4″, *J* = 5.2 Hz, *J* = 3.6 Hz), 7.77 (dd, 1H, H-5″, *J* = 4.8 Hz, *J* = 1.2 Hz), 7.99 (dd, 1H, H-3″, *J* = 3.6 Hz, *J* = 1.2 Hz), 8.14 (d, 2H, H-2, H-6, *J* = 8.4 Hz), 8.30 (d, 1H, H-4′, *J* = 9.2 Hz), 8.35 (d, 1H, H-5′, *J* = 8.8 Hz), 8.48 (d, 2H, H-3, H-5, *J* = 8.4 Hz), 10.19 (s, 1H, CHO) ppm. ^13^C-NMR (Acetone-*d*_6_, 400 MHz) *δ* 123.6, 125.8, 128.1, 128.2, 129.2, 130.6, 130.8, 138.3 141.6, 142.6, 155.1, 157.2, 192.6 ppm. MS (EI) *m*/*z* (%) = 267 ([M + 1]^+^, 5), 266 ([M]^+^, 30), 238 (4), 129 (5), 108 (100). HRMS: *m*/*z* (EI) for C_15_H_10_N_2_OS; calcd 266.0514; found 266.0512.

4-(6′-(Thiophen-2″-yl)pyridazin-3′-yl)benzonitrile **3d**. Yellow solid (28%). Mp: 249–252 °C. UV (ethanol): λ_max_ nm (*ε*, M^−1^·cm^−1^) 323, (26,086). IR (liquid film) *ν* 2,233 (C≡N) cm^−1^. ^1^H-NMR (Acetone-*d*_6_, 400 MHz) *δ* 7.29 (dd, 1H, H-4″, *J* = 5.2 Hz, *J* = 3.6 Hz), 7.76 (dd, 1H, H-5″, *J* = 5.0 Hz, *J* = 1.2 Hz), 7.98 (dd, 1H, H-3″, *J* = 3.8 Hz, *J* = 1.2 Hz), 8.00 (d, 2H, H-2, H-6, *J* = 8.8 Hz), 8.29 (d, 1H, H-5′, *J* = 9.2 Hz), 8.34 (d, 1H, H-4′, *J* = 9.2 Hz), 8.46 (d, 2H, H-3, H-5, *J* = 8.8 Hz) ppm. ^13^C-NMR (Acetone-*d*_6,_ 400 MHz) *δ* 114.1, 119.1, 123.7, 125.7, 128.2, 128.3, 129.2, 130.7, 133.6, 141.5, 155.3, 156.7, 206.1 ppm. MS (EI) *m*/*z* (%) = 264 ([M + 1]^+^, 5), 263 ([M]^+^, 31), 127 (6), 109 (6), 108 (100). HRMS: *m*/*z* (EI) for C_15_H_9_N_3_S; calcd 263.0517; found 263.0516.

3-(3′-Nitrophenyl)-6-(thiophen-2″-yl)pyridazine **3e**. Yellow solid (25%). Mp: 245–248 °C. UV (ethanol): λ_max_ nm (*ε*, M^−1^·cm^−1^) 314, (24,105). IR (liquid film) *ν* 3409, 2094, 1639, 1524, 1444, 1402, 1351, 1311, 1276, 1111, 1081, 1056, 838, 812, 727 cm^−1^. ^1^H-NMR (Acetone-*d*_6,_ 300 MHz) *δ* 7.29 (dd, 1H, H-4″, *J* = 5.2 Hz, *J* = 3.6 Hz), 7.78 (dd, 1H, H-5″, *J* = 5.2 Hz, *J* = 0.8 Hz), 7.92 (t, 1H, H-5′, *J* = 8.2 Hz), 8.00 (dd, 1H, H-3″, *J* = 3.2 Hz, *J* = 1.2 Hz), 8.33 (d, 1H, H-5, *J* = 9.2 Hz), 8.42 (dd, 1H, H-6′, *J* = 8.0 Hz, *J* = 1.2 Hz), 8.43 (d, 1H, H-4, *J* = 9.2 Hz), 8.70 (dd, 1H, H-4′, *J* = 7.8 Hz, *J* = 0.8 Hz), 9.10 (t, 1H, H-2′, *J* = 1.8 Hz) ppm. ^13^C-NMR (Acetone-*d*_6_, 300 MHz) *δ* 122.1, 123.8, 125.1, 125.6, 128.2, 129.2, 130.7, 131.3, 133.5, 139.0, 141.4, 149.8, 155.4, 156.2 ppm. MS (EI) *m*/*z* (%) = 284 ([M + 1]^+^, 4), 283 ([M]^+^, 47), 253 (27), 117 (70), 108 (100). HRMS: *m*/*z* (EI) for C_14_H_9_N_3_O_2_S; calcd 283.0415; found 283.0414.

#### 3.2.3. Nonlinear Optical Measurements

Hyper-Rayleigh scattering (HRS) was used to measure the orientationally averaged first hyperpolarizability *β* of the push–pull chromophores **3a**–**e**. The experimental set-up for HRS measurements is identical to that described in detail in reference [73].

Following reference [72] we have chosen to report our values using the so-called T (Taylor expansion) convention. Taking into account the most recent hyper-Rayleigh scattering measurement from CCl_4_ signal which was used as a reference [77], the corrected reference value for the first hyperpolarizatibity tensor element *β*_333_ of *p*NA in dioxane at 1064 nm is 40 × 10^−30^ esu. The standard two-level model, that ignores damping, was used to estimate the magnitude of the static first-order hyperpolarizability *β*_0_ [74,75,76]. Given the model’s simplicity, these extrapolated values should be viewed with caution.

#### 3.2.4. Theoretical Calculations

All theoretical calculations were performed in Gaussian 09 (Gaussian, Inc., Wallingford CT, USA, 2010) [78]. The geometry of individually molecule was optimized by the density functional theory (DFT) at the B3LYP level by employing the 6-311G** basis set and using polarizable continuum model using dioxane as the solvent. [keyword: SCRF = (PCM, Solvent = 1,4-Dioxane)]. Frequency calculations were achieved in order to ensure the absence of negative frequencies. Hyperpolarizability factors were estimated at the same level of theory using an incident wavelength of 1064 nm (keywords: freq = raman, cphf = rdfreq, polar) and with a polarized solvent continuum model using dioxane as the solvent.

## 4. Conclusions

A series of novel thienylpyridazines were prepared through palladium catalyzed Suzuki-Miyaura cross-coupling in low yields due to the possibility of competitive secondary reactions. The new molecules were functionalized with different electron acceptor groups, and the structures were confirmed by standard spectroscopic techniques.

All compounds exhibited strong and broad absorption bands that showed bathochromic shifts with the increase of the electron donating and electron accepting abilities of the donor/π-bridge and the electron-withdrawing group, respectively. All thienylpyridazines showed very weak emissive properties.

The potential of the synthesized thienylpyridazines as second harmonic generators was evaluated by hyper-Rayleigh scattering showing an enhancement of the hyperpolarizability *β* with the increase of the auxiliary electron donating ability of the donor group/π-spacer, with highest measured hyperpolarizability value being achieved by the thienylpyridazine functionalized with 4-cyanophenyl group (*β* = 175 × 10^−30^ esu).

DFT calculations were also carried out, showing coplanarity between the thienylpyridazine part of the molecule with the formyl- thiophene or furan end-cap, reduced planarization with the phenyl-based substituents, and generally diffuse and overlapping HOMO and LUMO densities. The estimated dipole moments for the five molecules range between 4.3 and 8.9 Debye (in 1,4-dioxane), and exhibit a higher correlation with the experimentally determined hyperpolarizabilities than the calculated hyperpolarizabilities.

## Figures and Tables

**Table 1:** Yields, UV-Visible absorption and emission data for thienylpyridazines **3a**–**e**.

Cpds.	Yield	Absorption ^1^		Emission ^2^		
		λ_max_ (nm)	*ε* (M^−1^·cm^−1^)	λ_em_ (nm)	Stokes’ Shift (cm^−1^)	*Φ_F_*
**3a**	28	357	29,800	428	4650	0.006
**3b**	14	354	27,800	432	5100	0.006
**3c**	15	332	26,000	405	5430	0.003
**3d**	28	323	26,100	404	6210	0.004
**3e**	25	314	24,100	408	7340	0.005

^1^ Absorption spectra were recorded in ethanol solutions, at room temperature. ^2^ Emission spectra were recorded in ethanol solutions, at room temperature, using a solution of quinine sulfate in 0.05 M H_2_SO_4_ as a quantum yield standard (*Φ_F_* = 0.51 for excitation at 347 nm) [66,67].

**Table 2:** UV-Visible absorption, *β* values, *β*_0_ values for thienylpyridazines **3a**–**e** and *p*-nitroaniline reference.

Cpds.	Absorption ^1^		*β* ^2^ (10^−^^30^ esu)	*β*_0_^3^ (10^−^^30^ esu)
	λ_max_ (nm)	*ε* (M^−1^·cm^−1^)		
**3a**	348	24,100	155	75
**3b**	348	31,100	100	50
**3c**	326	24,900	54	30
**3d**	323	26,100	175	100
**3e**	314	24,100	-	-
***p*NA**	370	-	62	28

^1^ Absorption data and experimental first hyperpolarizabilities *β* were measured in 1,2-dioxane solution. ^2^ All compounds are transparent at the 1064 nm fundamental wavelength and the hyperpolarizability values are reported using the T-convention [77]. ^3^ Data corrected for resonance enhancement at 532 nm using the two-level model with *β*_0_ = *β* [1 − (λ_max_/1064)^2^][1 − (λ_max_/532)^2^]; damping factors not included 1064 nm.

**Table 3:** Computational results summary of thienylpyridazines **3a**–**e** hyperpolarizability calculations.

Cpds.	*μ* (D) ^1^	*β_II_* (10^−30^ esu) ^2^	*β_tot_* (10^−30^ esu) ^3^	E_HOMO_ (eV) ^4^	E_LUMO_ (eV) ^5^	E_g_ (eV) ^6^
3a	8.98	53.17	88.61	−6.27	−2.73	3.54
3b	5.56	42.63	70.97	−6.24	−2.63	3.61
3c	5.33	56.38	93.89	−6.35	−2.56	3.79
3d	8.04	41.48	69.06	−6.40	−2.50	3.90
3e	4.34	29.07	48.33	−6.41	−2.79	3.62

^1^ Dipole moment; ^2^ Parallel hyperpolarizability; ^3^ Total hyperpolarizability; ^4^ Energy of the Highest Occupied Molecular Orbital; ^5^ Energy of the Lowest Unoccupied Molecular Orbital. ^6^ LUMO-HOMO energy gap.
